# Primary spindle cell tumor originating from the liver that was difficult to diagnose

**DOI:** 10.1186/s40792-022-01530-6

**Published:** 2022-09-19

**Authors:** Tomohiko Ikehara, Tadaaki Shimizu, Shohei Hirano, Kentaro Fukushima, Jun-ichi Yoshizawa, Toshitsugu Nakamura, Ataru Nakayama

**Affiliations:** 1 Department of Surgery, Ina Central Hospital, 1313-1, Koshirokubo, Ina, Nagano, 396-8555 Japan; 2 Department of Pathology, Ina Central Hospital, 1313-1, Koshirokubo, Ina, Nagano, 396-8555 Japan

**Keywords:** Spindle cell tumor, Liver, Hepatectomy, Pathology, Immunostaining

## Abstract

**Background:**

It has been reported that hepatocellular carcinoma (HCC) with spindle cell tumor accounts for 1.8% of all HCCs, but spindle cell tumors that do not show an obvious conventional HCC are extremely rare. In this report, we describe a case of resection of a primary spindle cell tumor of the liver that was difficult to diagnose.

**Case presentation:**

A 75-year-old man presented with fever and right chest pain. He was suspected of a giant primary diaphragmatic tumor of extrahepatic origin by imaging studies. The preoperative differential diagnosis included benign masses such as myxoid sarcoma and schwannoma, and we planned a diaphragmatic resection. Intraoperatively, however, dissection of the tumor from the liver was not possible, requiring an extended right posterior segmentectomy with combined resection of the diaphragm. The patient had a good postoperative course and 1 year has passed since the surgery with no recurrence. The pathology showed that the mass was located just below the hepatic capsule/parenchymal region and was adherent to the diaphragm, but there was no continuity. The morphology suggested a low-grade mesenchymal tumor such as a solitary fibrous tumor and perivascular epithelioid cell tumor, but immunostaining was negative, making the diagnosis difficult. Although some areas of high proliferative activity were observed, finally, the diagnosis of primary spindle cell tumor of the liver with smooth muscle differentiation was made based on the positive results of muscle markers such as αSMA, desmin, and h-caldesmon.

**Conclusions:**

Spindle cell tumor arising from the liver is so rare that preoperative and pathological diagnosis is often difficult to reach. Although further studies are needed to elucidate and better understand this uncommon clinical entity, we consider that complete resection is necessary for the above case, which may contribute to long-term survival.

## Background

Spindle cell carcinoma, a subtype of hepatocellular carcinoma (HCC), usually consists of epithelial components of HCC intermingled with pseudosarcomatous areas [1], and this partial spindle cell transformation is also seen in many other epithelial tumors, such as those of the upper digestive tract [2], skin [3], breast [4], female genital tract [5], urinary tract [2], and lung [6, 7]. However, those consisting only of spindle cells are most likely not derived from hepatocytes, and cases without sarcomatous change are extremely rare [8]. We herein report a patient with a primary spindle cell tumor of the liver that was difficult to diagnose, who successfully underwent extended hepatectomy.

## Case presentation

**Fig. 1:**
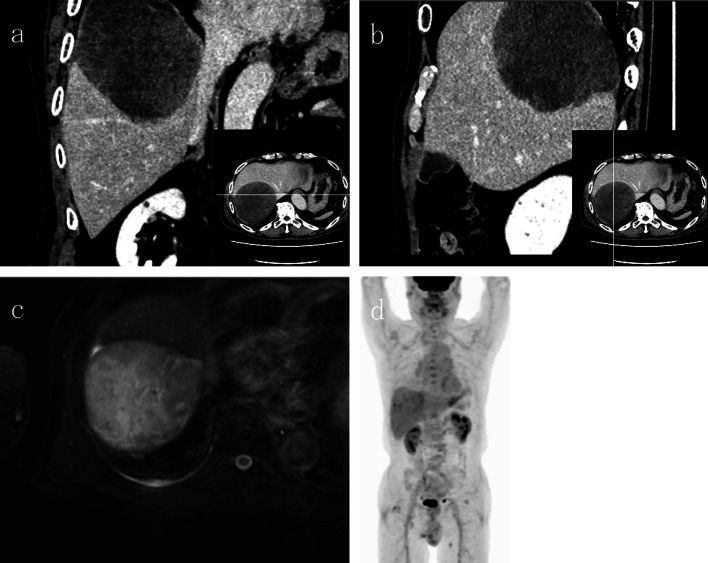
Contrast-enhanced computed tomography (CT), contrast-enhanced magnetic resonance imaging (MRI), and positron emission tomography (PET). **a**, **b** CT shows a well-circumscribed and smooth-marginated, huge 10-cm mass under the right diaphragm. The main feeder for the tumor is the right inferior phrenic artery. **c** MRI demonstrates heterogeneous high signal intensity on T2WI. **d** PET revealed abnormal uptake in the tumor with a SUVmax of 4.6

**Fig. 2:**
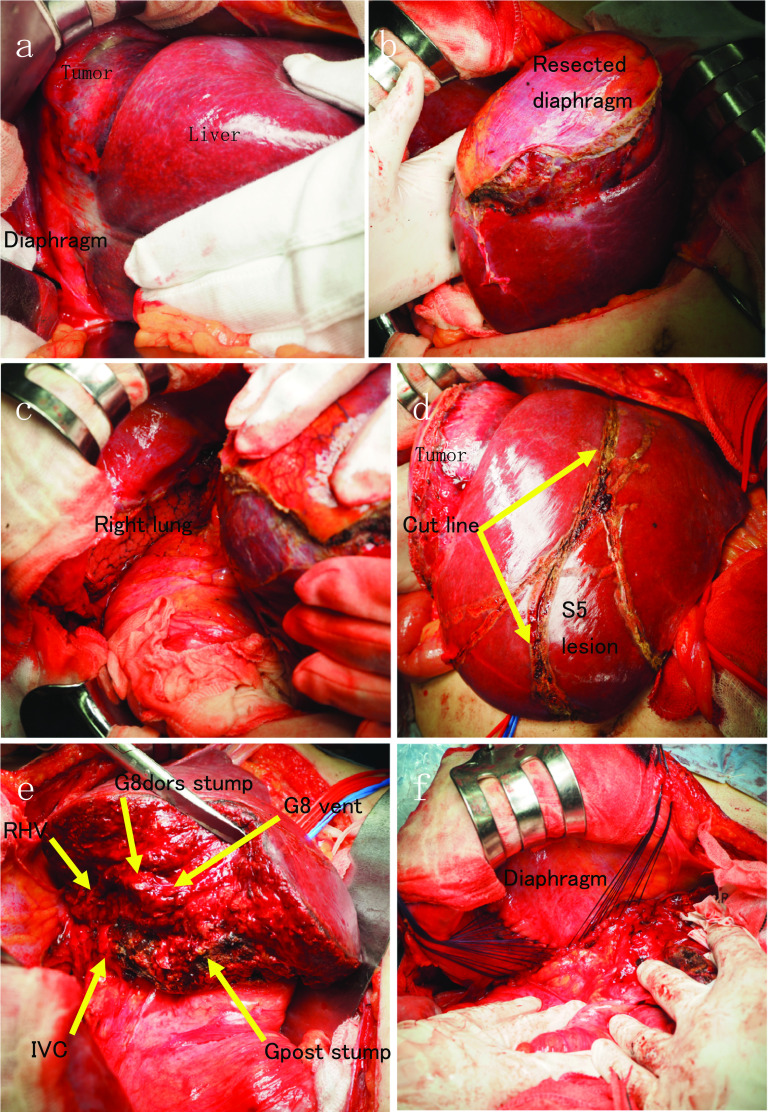
Surgical procedure. **a** Separation of the tumor from the liver was not possible. **b**, **c** Combined resection of the diaphragm was performed. **d** By clamping A5, we found the boundary between S5 and S6. **e** We performed extended right posterior segmentectomy (S6, 7, 8dors resection). **f** simple closure of the diaphragm was possible

**Fig.3:**
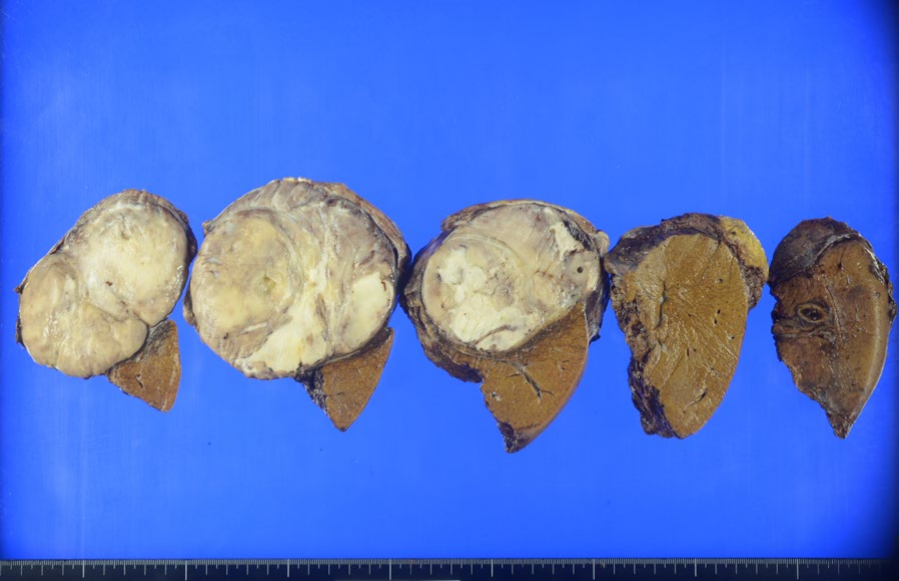
Macroscopic of the resected specimen. A well-circumscribed nodular tumor of 11 × 10 × 8.5 cm in size was located just below the hepatic capsule/intraparenchymal space and was adherent to the diaphragm, but there was no continuity with the diaphragm

**Fig. 4:**
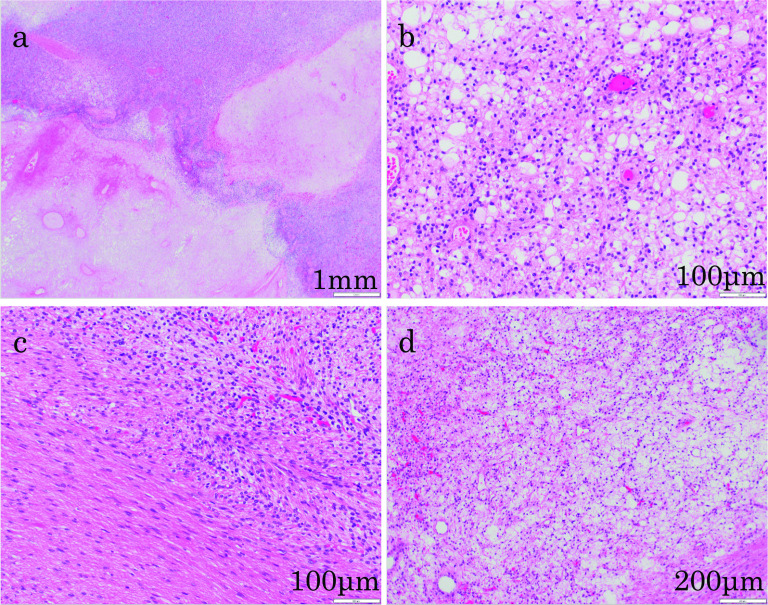
Microscopic findings of the tumor. Hematoxylin and eosin staining. **a** Section shows a proliferation of anaplastic cells with clear cytoplasm and round heteroplasm. Map-like necrotic foci are seen in some areas. **b** Some areas contain fat droplets and exhibit lipomatous features. Mitotic figures are rarely seen. **c** Bundled proliferation of spindle-shaped cells is seen in some tumors. **d** Myxoid or microcystic changes of the stroma and hemangiopericytomatous vessels are also seen

**Fig. 5:**
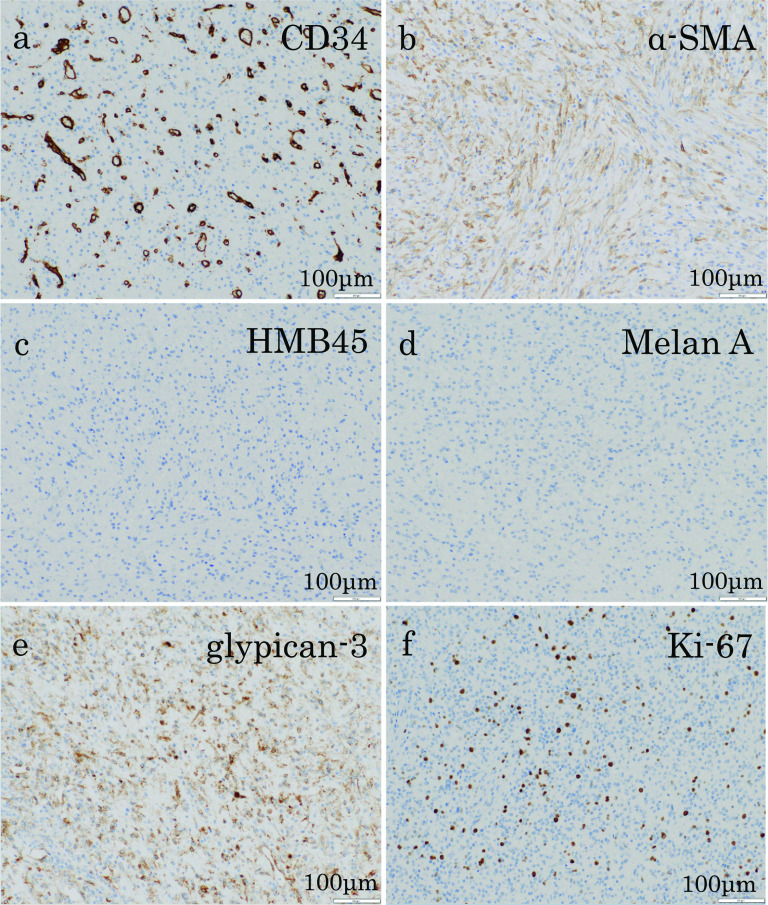
Immunohistochemistry. **a**, **c**, **d** The tumor cells are negative for CD34, HMB45, Melan A. **b**, **e** The tumor cells are positive for α-SMA, glypican-3.** f** Ki-67 index is 10% (hot spot)

A 75-year-old man presented with fever and right chest pain. The blood biochemical tests showed the following: lactate dehydrogenase (LDH), 251 IU/L; alkaline phosphatase (ALP), 469 IU/L; gamma-glutamyl transferase (γ-GTP), 125 IU/L; creatinine (Cre) 1.43 mg/dL; C-reactive protein (CRP) 3.04 mg/dL; squamous cell carcinoma-related antigen (SCC), 1.8 ng/mL; nerve specific enolase (NSE), 21.6 ng/mL; soluble interleukin-2 receptor (SIL-2R), 614 U/mL. Computed tomography (CT) showed a huge 10-cm mass under the right diaphragm (Fig. 1a, b). This well-circumscribed and smooth-marginated tumor showed minor enhancement in the lower density areas close to the fat and showed a gradual, heterogeneous mild enhancement of tumor and liver margins. The main feeder for the tumor is the right inferior phrenic artery, and this tumor was supposed to be a sarcomatoid malignancy originating from the diaphragm rather than a benign disease such as schwannoma on CT. No findings were suggesting obvious pulmonary invasion. Magnetic resonance imaging (MRI) demonstrated heterogeneous high signal intensity on T2-weighted images (T2WI) and diffusion-weighted images (DWI) (Fig. 1c). Contrast-enhanced MRI showed gradual heterogeneous enhancement similar to CT findings. Findings of extrahepatic development were also obtained, but the possibility of adhesion to the liver was suspected. Fluorodeoxyglucose-positron emission tomography (FDG-PET) revealed abnormal uptake in the tumor with a maximal standardized uptake value (SUVmax) of 4.6 (Fig. 1d). The uptake was not so high, and a myxoid type sarcoma, such as myxoid liposarcoma, or benign tumor was considered. Although a definitive diagnosis could not be achieved, we scheduled a diaphragmatic resection for suspicion of benign tumors of the diaphragm, such as mucous-type sarcoma or schwannoma. However, we performed extended right posterior segmentectomy with combined resection of the diaphragm, because separation of the tumor from the liver was not possible and the tumor was close to the posterior Glisson’s pedicles and segment 8 dorsal Glisson’s pedicles (Fig. 2a–e). The total operation time was 8 h 10 m, and the total blood loss was 1459 mL. Although the major diameter of the diaphragmatic defect was 12 cm, simple closure was possible (Fig. 2f). The patient’s postoperative course was uneventful, and he was discharged 14 days after surgery. Pathological findings showed that the mass was located just below the hepatic capsule/intraparenchymal space and was adherent to the diaphragm, but there was no continuity with the diaphragm (Fig. 3). Mitosis was unremarkable, about 0–1 cells/10 HPF. The morphology suggested low-grade mesenchymal tumors such as solitary fibrous tumor (SFT) and perivascular epithelioid cell tumor (PEComa) (Fig. 4a–d), but immunostaining was negative for these tumors, making the diagnosis difficult (Fig. 5a–f). We diagnosed the tumor as a spindle cell tumor with smooth muscle differentiation because of the positive results of myosin markers such as αSMA, desmin, and h-caldesmon, although some areas with high proliferative activity were observed. 1 year has passed since the surgery with no recurrence.

## Discussion

Spindle cell tumors, first described by Weiss and Enzinger in 1896, are a small subset of mesenchymal cell tumors that arise primarily from mesodermal tissue [9]. It includes both benign and malignant tumors, with the former subclass including schwannomas, neurofibromas, leiomyomas, and fibromas. However, most cases belong to the intermediate group and are difficult to classify, and malignant transformation of benign tumors is often encountered [10]. Spindle cell tumors are derived from various types of tumors such as fibrosarcoma, gastrointestinal stromal tumors, and intra-abdominal desmoid tumors [11], and in malignant cases, carcinoma, an epithelial malignancy, and sarcoma, a stromal malignancy, are found within the same tumor [12, 13]. No common view of the histological features has been established in the stromal area, which is mainly composed of spindle-shaped cells that constitute the sarcoma-like component [14]. The WHO officially classified spindle cell tumors as soft tissue neoplasms in 1994, and neoplasms with spindle cells are rarely seen in malignant tumors of various organs, with reported frequencies of 2.2% [15], 3.1% [16], 0.3% [17], and 0.4% [18] in cancers of the esophagus, thyroid, lung, and mammary gland, respectively.

In the liver, the proportion of hepatocellular carcinoma with spindle cells is estimated to be 1.8% of all resected hepatocellular carcinomas [19] and is usually observed as partial spindle cellularity with the epithelial component of the hepatocellular carcinoma intermingled with pseudosarcomatous areas [1]. This is a so-called sarcomatoid carcinoma, which often shows a transition pattern between the carcinoma and sarcoma components, resulting in a sarcomatoid transformation of some of the carcinoma components [20], or a pattern in which stem cells can differentiate into both epithelial and non-epithelial cells become carcinoma [21]. On the other hand, reports of tumors with spindle cell tumor images that do not clearly show the usual type of hepatocellular carcinoma are extremely rare, and as far as we could find, only two cases [8] were reported, including our case. The giant liver tumor reported by Kato et al. was composed of spindle-shaped cells with no epithelial tumor appearance and showed sarcoma-like changes, but no obvious epithelial elements were detected except for the positive keratin, so the tumor was classified as a primary malignant spindle cell tumor of the liver considering its unknown origin.

Histologically, our case was suspected to have a low-grade mesenchymal tumor such as SFT, PEComa, or liposarcoma, but there was no noticeable change in the sarcomatoid component, and immunostaining was negative for melanocyte markers, CD34, STAT6, and CDK4, then there was little evidence to support these findings. It was difficult to diagnose the tumor, but we concluded that it was a primary spindle cell tumor of the liver showing smooth muscle differentiation because of the positivity of three muscle system markers (Table 1). Currently, there is no subclassification of spindle cell tumor in the WHO classification of liver tumors, but the number of tumor types is increasing with time, and the accumulation of such cases in the future may suggest the establishment of a subclassification similar to that of breast and lung tumors.

**Table 1:** Immunohistochemical findings

Positive	Negative
α-SMA	CD34
Desmin (very focal)	Melan A
h-Caldesmon (very focal)	HMB45
MDM2 (focal)	bcl-2
P16 (focal)	STAT6
EMA (very focal)	DOG1
AE1/AE3 (very focal)	CDK4
Glypican-3	S100

Although the imaging characteristics of spindle cell tumors have not been established [22] and differential diagnosis is generally difficult [23], a needle biopsy may be considered as an aid in improving preoperative diagnosis. However, it should be noted that the accuracy rate of the preoperative diagnosis is not high [24, 25] and may be a cause of dissemination [26], so it may not be recommended aggressively.

The feeding artery of the tumor, in our case, was the right inferior phrenic artery, and it was diagnosed preoperatively as a primary diaphragmatic tumor with a predominantly expansive growth. However, intraoperative findings could not determine whether the liver or the diaphragm was the primary tumor, so a hepatic resection with combined resection of the diaphragm was performed to remove the tumor, and an R0 resection was obtained. Finally, the pathological diagnosis was that of a tumor originating from the liver, and the difficulty of preoperative diagnosis was apparent. Therefore, although there was no continuity between the tumor and the diaphragm histologically, diaphragmatic invasion of the liver tumor was considered clinically. Extrahepatic development of liver tumors is rare, but has been reported mainly in Japan, and the mechanisms of HCC with such characteristics include (1) carcinomatosis of the accessory hepatic lobe; (2) carcinomatosis of ectopic liver tissue; (3) development from the Riedel lobe; (4) development from the cirrhotic protrusion, and (5) extrahepatic progress of marginal liver cancer [27–29]. Although this case was not HCC, it was considered to be consistent with (5) by process of elimination. Furthermore, Miyagawa et al. [30] noted that the presence of extrahepatic feeding vessels and large tumor diameter are significant factors contributing to multiorgan invasion in extrahepatic growth-type HCC, and concluded that the possibility of multiorgan invasion should be considered when extrahepatic feeding vessels are found in a large extrahepatic HCC. Among such tumors, two cases [31] have been reported in which it was difficult to distinguish them from diaphragmatic tumors, and only one case [32], in which the right inferior phrenic artery was the main feeder, as in the present case, was extremely rare. Although the histological findings in our case were not positively suggestive of malignancy, the ki-67 positivity rate was higher at the margins of the extrahepatic tumor than at other sites, indicating high proliferative activity. The tumor cells that originated in the hepatic capsule or its vicinity developed extrahepatic growth with somewhat higher proliferative activity, and the right inferior phrenic artery was predominantly observed as the main feeder, which histologically supported the above-mentioned characteristics of extrahepatic liver tumors with multiple organ involvement.

Maeda et al. [19] and Jernigan et al. [33] stated that HCC subtypes with spindle cell components have very aggressive biology and often cause portal vein invasion or extrahepatic metastasis, and therefore have a poor prognosis [34]. However, Kaibori et al. [35] reported that the prognosis of the resected group was significantly better than that of the unresected group in cases of primary sarcomatoid carcinoma of the liver (P = 0.001). In addition, there are some reports that long-term survival can be expected by complete resection even in cases of primary liver liposarcoma [36], which tends to show extracapsular invasion [37]. In the current situation where no other treatment methods have been established, tumor resection should be performed aggressively for the primary spindle cell tumor of the liver. Since this case is not as malignant as sarcoma or liposarcoma and is considered to be different from them, there is not sufficient evidence at this time to conclude that partial resection rather than anatomical resection is acceptable even for peripheral lesions. As to which of the two techniques is more appropriate, it may be controversial, as in HCC, but in accordance with the extrahepatic development of HCC discussed above, anatomical resection may be the choice to be made whenever possible in the past literature [38, 39]. Although the patient has been recurrence-free for one year after complete resection, strict follow-up is necessary for the future, and it is important to continue accumulating and examining this kind of case to establish other treatments such as adjuvant therapy.

## Conclusions

We reported a case of a spindle cell tumor originating from the liver. Spindle cell tumor arising from the liver is so rare that preoperative and pathological diagnosis is often difficult to reach. Complete resection should be considered in cases where the above disease is the differential diagnosis and may contribute to long-term survival.

## Data Availability

All data generated during this study are included in this published article.

## Abbreviations

HCC: Hepatocellular carcinoma
CT: Computed tomography
MRI: Magnetic resonance imaging
T2WI: T2-weighted images
DWI: Diffusion-weighted images
FDG-PET: Fluorodeoxyglucose-positron emission tomography
SFT: Solitary fibrous tumor
PEComa: Perivascular epithelioid cell tumor
